# Prospective application of a prediction model for lateral lymph node metastasis in papillary thyroid cancer patients with central lymph node metastasis

**DOI:** 10.3389/fendo.2023.1283409

**Published:** 2024-01-04

**Authors:** Yunhan Ma, Yi Li, Luming Zheng, Qingqing He

**Affiliations:** ^1^ Department of General Surgery, The 960th Hospital of the PLA Joint Logistics Support Force, Jinan, China; ^2^ Jinzhou Medical University, Jinzhou, Liaoning, China

**Keywords:** papillary thyroid carcinoma, lateral lymph node metastasis, central lymph node metastasis, prediction mode, clinical lymph node-negative (cN0)

## Abstract

**Objective:**

This study aimed to develop and apply a prediction model to estimate the probability of lateral lymph node metastasis (LLNM) in patients with cN0 unilateral papillary thyroid carcinoma (PTC) with central lymph node metastasis (CLNM).

**Setting:**

All study data were collected from a single tertiary hospital.

**Methods:**

Univariable and multivariable logistic regression analyses were used to explore independent predictors of LLNM in the derivation and internal validation cohorts, which were used to construct and validate a nomogram. Another 96 patients were included prospectively to evaluate the efficacy of this nomogram.

**Results:**

Maximum tumor diameter greater than 1.0 cm (OR, 2.712; 95% CI, 1.412–5.210), multifocality (OR, 2.758; 95% CI, 1.120–6.789), the number of CLNM ≥3 (OR, 2.579; 95% CI, 1.315–5.789), CLNM ratio ≥0.297 (OR, 2.905; 95% CI, 1.396–6.043), and tumors located in the upper portion (OR 2.846, 95% CI 1.151–7.039) were independent predictors associated with LLNM. The prediction model showed excellent discrimination with an AUC of 0.731 (95% CI, 0.635–0.827). Novel risk stratification for LLNM was constructed based on this nomogram. In the prospective cohort, we stratified these patients into three risk subgroups: low-, moderate-, and high-risk subgroups and we found that the probability of LLNM was positively correlated with the total points from the nomogram.

**Conclusion:**

This nomogram was applied in prospective clinical practice and distinguished PTC patients with a genuinely high risk of LLNM. Surgeons can use our nomogram to tailor the surgical plan and to credibly determine further postoperative therapy.

## 1 Introduction

Thyroid cancer (TC) is a common endocrine malignancy with a sharp increase in incidence in China (1, 2). Papillary thyroid carcinoma (PTC) is the major pathological type of TC and has a favorable prognosis (3). Although PTC-specific mortality at 10 years is less than 5%, patients with locally advanced PTC or cervical lymph node metastasis (LNM) still face a high risk of recurrence (4, 5).

The incidence rate of LNM in PTC patients ranges from 40% to 90% (6, 7). LNM develops in a stepwise manner (8). The central lymph nodes (CLNs) on the ipsilateral side of the thyroid tumor are the first compartment for routine LNM, namely, central lymph node metastasis (CLNM), followed by ipsilateral lateral lymph node metastasis (LLNM) through lymphatic drainage (9). For clinically lymph node negative (cN0) PTC, the indications for lymph node dissection (LND) are still controversial. According to the American Thyroid Association (ATA) management guidelines (10) and National Comprehensive Cancer Network (NCCN) Guidelines for TC (www.nccn.org/guidelines), prophylactic central lymph node dissection (pCLND) is not recommended, although endocrine surgeons in Japan recommend routine pCLND (11). The sensitivity of preoperative assessment is relatively low, and the incidence of occult CLNM ranges from 30% to 80% in cN0 patients (12). Notably, Chinese PTC patients have a much higher incidence of occult CLNM than other populations (13), and some experts (14) recommend pCLND based on associated research. Occult LLNM is believed to be an independent risk factor for recurrence and reoperation (15). Therapeutic lateral lymph node dissection (LLND) should be performed in patients with clinically suspicious LLNM (cN1b) confirmed by preoperative examination and fine-needle aspiration biopsy (FNAB). Prophylactic lateral lymph node dissection (pLLND) is not recommended. Previous studies showed that the incidence of occult LLNM ranged from 18.6% to 64% (16, 17). Recurrence may occur rapidly in patients with occult LLNM who undergo thyroidectomy without pLLND (18). Therefore, identifying LLNM as accurately as possible is important for surgeons to make a surgical plan.

Nomogram-based prediction models have been widely applied for the diagnosis of various types of cancers, including PTC (19–21). In this study, we investigated the preoperative clinical data and intraoperative rapid frozen pathological characteristics to analyze their effects on LLNM risk. The first purpose of our study was to identify the independent risk factors for LLNM in patients with PTC and CLNM. The second purpose was to develop an effective prediction model for prospectively evaluating LLNM. To the best of our knowledge, few studies have applied prediction models to prospective cohort studies.

## 2 Materials and methods

### 2.1 Patient cohort

This study was approved by the 960th Hospital of the PLA Joint Logistics Support Force Research Ethics Committee (No. 2022-57) and registered in the Chinese Clinical Trial Registry (ChiCTR2200064277).Patients in the prospective validation cohort provided informed consent with complete understanding of the purpose before accepting the prediction and surgery.

**Figure 1:**
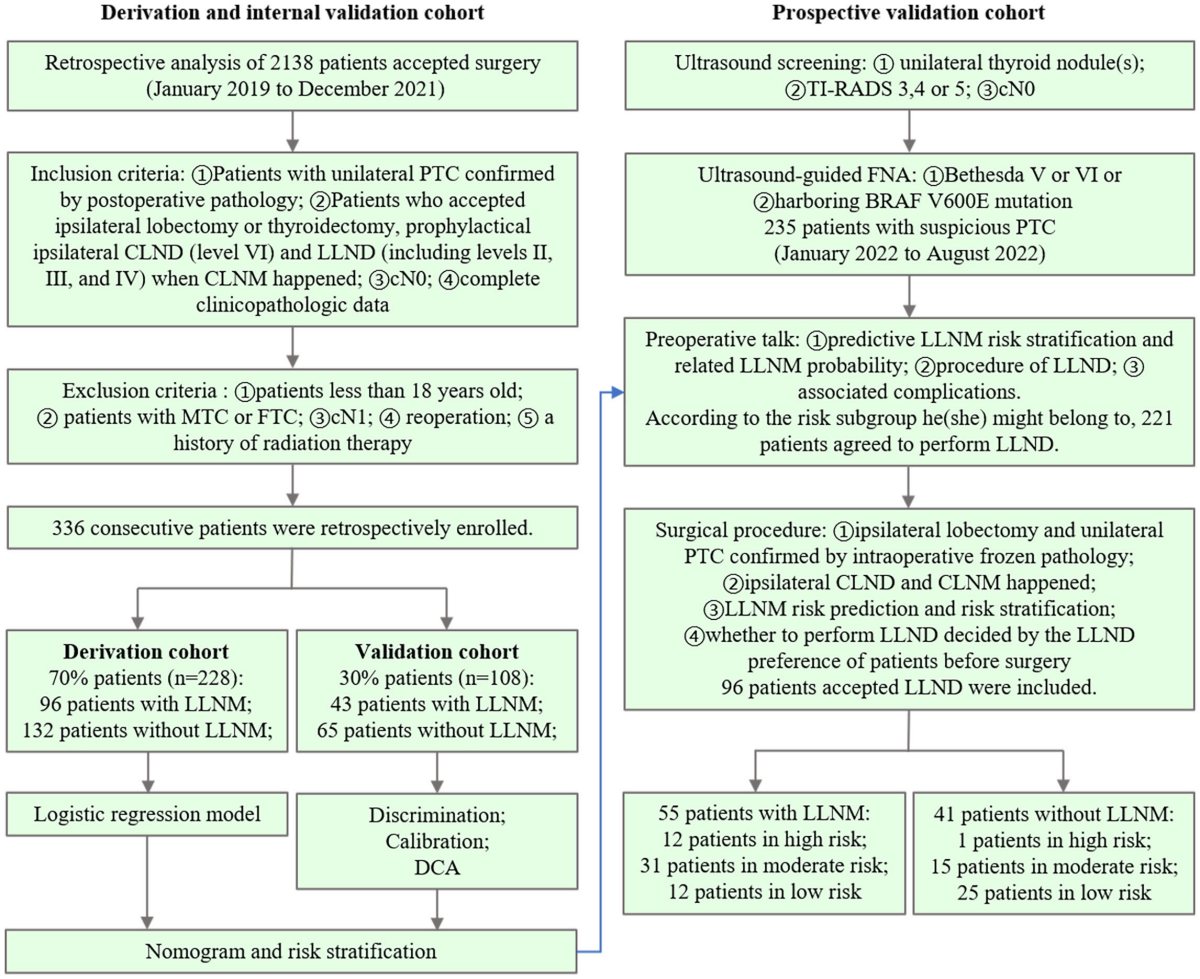
Patient inclusion and exclusion flowchart for the derivation and internal validation cohorts (left) and prospective validation cohorts (right), along with the study design used for each cohort.

**Figure 2:**
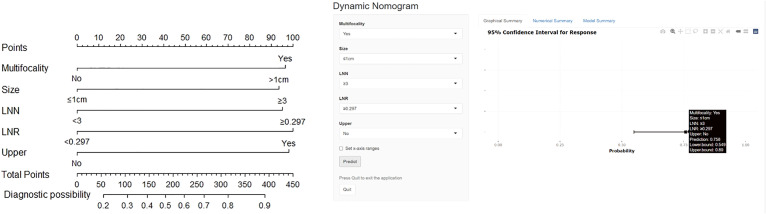
The nomogram for prediction of LLNM (left) and dynamic nomogram were used to stratify PTC patients intraoperatively (https://docliyi.shinyapps.io/dynnomapp/).

Surgical treatments are recommended for patients with Bethesda V or VI thyroid nodules or those harboring the BRAF V600E mutation (22). The inclusion criteria were as follows: (1) patients with unilateral PTC confirmed by postoperative pathology; (2) patients who underwent ipsilateral lobectomy, prophylactic ipsilateral CLND (level VI), and LLND (including levels II, III, and IV) when CLNM occurred; (3) cN0 (23), clinical and ultrasound examination did not find enlarged or swollen lymph nodes with obvious malignant signs before surgery; (4) and complete clinicopathologic data. (5) Patients who understood and accepted intraoperative rapid frozen pathology. The exclusion criteria were as follows: (1) age <18 years; (2) medullary thyroid carcinoma (MTC); (3) follicular thyroid carcinoma (FTC); (4) cN1 PTC; (5) reoperation; and (6) a history of radiation therapy. Between January 2019 and December 2021, 336 consecutive patients were retrospectively enrolled. We used simple randomization to divide the patients into derivation and internal validation cohorts at a ratio of 7:3 (**Figure 1**). A nomogram for predicting LLNM was constructed for these patients (**Figure 2**). Based on the total points from the nomogram, we stratified PTC patients into three subgroups: low-, moderate-, and high-risk LLNM (24).

To build a prospective validation cohort, 235 consecutive cN0 patients with unilateral suspicious PTC diagnosed with FNAB were initially selected from January 2022 to August 2022. The inclusion and exclusion criteria are detailed in the flowchart (**Figure 1**). Before surgery, we explained the predictive LLNM risk stratification and related LLNM probability, LLND procedure, and associated complications to patients in detail. Patients with unilateral PTC confirmed using intraoperative rapid frozen pathology underwent ipsilateral CLND. Intraoperative rapid frozen pathology of CLNs was also performed to determine the number and ratio of CLNM. Based on the constructed nomogram and intraoperative data, we quantitatively predicted the risk of LLNM and provided risk stratification for each patient. Only PTC patients with ipsilateral CLNM who received prophylactic LLND were included in the prospective validation cohort.

### 2.2 Surgical methods and pathological results

All patients with unilateral lesions underwent lobectomy according to specific indications and patient preferences. Ipsilateral pCLND was performed routinely, including prelaryngeal, pretracheal, and paratracheal CLNs. It is noteworthy that the right paratracheal CLNs included the posterior right recurrent laryngeal nerve CLNs. LLND was defined as compartment-oriented functional lateral neck dissection, including ipsilateral levels II to IV.

PTC pathology was classified according to the American Joint Committee on Cancer (AJCC) 8th guideline (25). CLNs removed by surgeons were assessed by intraoperative rapid frozen pathology and postoperative pathology, and LLNs were assessed only by postoperative pathology. The ratio of CLNM refers to the number of CLNM divided by all removed CLNs. For multifocal cases, we considered the diameter of the largest tumor as the maximum tumor diameter based on intraoperative rapid freezing results.

This study was in line with the STROCSS criteria (26).

### 2.3 Prediction model development and validation

We performed a univariate analysis to explore the differences in clinicopathological characteristics between the LLNM-positive and LLNM-negative groups. Variables with statistically significant differences from the univariate analysis were used as candidate variables for multivariate logistic regression to construct a nomogram. The appropriate cutoff values of maximum tumor diameter (cutoff = 1.0 cm), the number of CLNM (cutoff = 3) and the ratio of CLNM (cutoff = 0.297) were determined by receiver operating characteristic (ROC) analysis and the maximal Youden index.

The area under the curve (AUC) was calculated to evaluate the discriminative ability of the nomogram. Meanwhile, the calibration curve and Hosmer–Lemeshow test were used to reduce the overfitting bias and assess the goodness of fit by comparing the actual probabilities and the probabilities predicted by our nomogram. To estimate the net benefits of the nomogram model under different threshold probabilities, decision curve analysis (DCA) was performed. The sensitivity, specificity, positive predictive value (PPV), and negative predictive value (NPV) of the prediction model were also calculated.

### 2.4 Statistical analysis

Statistical analyses were implemented with the SPSS 26.0 (SPSS Inc., Chicago, IL, USA) and R software (v4.0.1). Statistical significance was set at P-value <0.05. Student’s t-test was used to analyze continuous variables that fit the normal distribution. Pearson’s chi-squared test or Fisher’s exact test were adopted to analyze categorical variables. Multivariate logistic regression analysis was conducted to estimate the odds ratio (OR) with a 95% confidence interval (95% CI) and identify independent predictor variables for LLNM. A nomogram was constructed, and its performance was evaluated using the ROC curve (“pROC” package), calibration curve (“RMS” package) and DCA (“rmda” package).

## 3 Results

### 3.1 Model development and internal validation

Patient characteristics are shown in **Table 1**. CLNM was observed in all the patients. The mean number of CLNM was 3.19 ± 2.89 and the mean ratio of CLNM was 0.34 ± 0.25. LLNM were confirmed to occur in 139 (41.4%) patients and the mean number of LLNM was 2.37 ± 1.90.

**Table 1:** The characteristics of patients.

Variables		Derivation cohort (n = 228)	Validation cohort (n = 108)	*P*-value	Retrospective cohort (n = 336)	Prospective cohort (n = 96)	*P*-value
Gender	Female	170 (69.3)	75 (30.7)	0.324	245 (76.3)	76 (23.7)	0.216
	male	58 (63.7)	33 (36.3)		91 (82.1)	20 (17.9)	
Age	<55 years	200 (70.0)	86 (30.0)	0.054	286 (78.4)	79 (21.6)	0.500
	≥55 years	28 (56.0)	22 (44)		50 (74.6)	17 (25.4)	
HT	No	185 (69.2)	82 (30.8)	0.372	267 (78.8)	72 (21.2)	0.348
	Yes	43 (62.3)	26 (37.7)		69 (74.2)	24 (25.8)	
Calcification	No	206 (67.5)	99 (32.5)	0.697	305 (77.8)	87 (22.2)	0.965
	Yes	22 (71.0)	9 (29.0)		31 (77.5)	9 (22.5)	
Maximum tumor diameter	≤1 cm	146 (70.2)	62 (29.8)	0.224	208 (79.1)	55 (20.9)	0.414
	>1 cm	82 (64.1)	46 (35.9)		128 (75.7)	41 (24.3)	
Upper portion	No	197 (67.2)	96 (32.8)	0.320	293 (80.0)	73 (20.0)	**0.007**
	Yes	31 (72.1)	12 (27.9)		43 (65.2)	23 (34.8)	
BRAFV600E	No	81 (66.4)	41 (33.6)	0.665	122 (77.7)	35 (22.3)	0.979
	Yes	147 (68.7)	67 (31.3)		214 (77.8)	61 (22.2)	
Nodular goiter	No	63 (65.5)	33 (34.5)	0.580	96 (71.1)	39 (28.9)	**0.025**
	Yes	165 (68.8)	75 (31.2)		240 (80.8)	57 (19.2)	
Multifocality	No	195 (67.9)	92 (32.1)	0.934	287 (79.3)	75 (20.7)	0.087
	Yes	33 (67.3)	16 (32.7)		49 (70.0)	21 (30.0)	
ETE	No	202 (68.9)	91 (31.1)	0.324	293 (80.5)	71 (19.5)	**0.002**
	Yes	26 (60.5)	17 (39.5)		43 (63.2)	25 (36.8)	
Prelaryngeal LNM	No	199 (68.9)	90 (31.1)	0.331	289 (81.0)	68 (19.0)	**0.001**
	Yes	29 (61.7)	18 (38.3)		47 (62.7)	28 (37.3)	
Pretracheal LNM	No	115 (72.3)	44 (27.7)	0.097	159 (82.8)	33 (17.2)	**0.024**
	Yes	113 (63.8)	64 (36.2)		177 (73.8)	63 (26.2)	
Paratracheal LNM	No	42 (64.6)	23 (35.4)	0.534	65 (73.9)	23 (26.1)	0.322
	Yes	186 (68.6)	85 (31.4)		271 (78.8)	73 (21.8)	
Number of CLNM ≥3	No	128 (68.4)	59 (34.4)	0.518	187 (81.3)	43 (18.7)	0.064
	Yes	100 (67.1)	49 (32.9)		149 (73.8)	53 (26.2)	
Ratio of CLNM ≥0.297	No	126 (67.0)	62 (33)	0.743	188 (81.4)	43 (18.6)	0.053
	Yes	102 (68.9)	46 (31.1)		148 (73.6)	53 (26.4)	
LLNM	No	132 (67.0)	65 (33.0)	0.691	197 (81.7)	44 (18.3)	**0.026**
	Yes	96 (69.0)	43 (31.0)		139 (72.8)	52 (27.2)	

HT, Hashimoto thyroiditis; ETE, extrathyroidal extension; CLNM, central lymph node metastasis; LLNM, lateral lymph node metastasis; upper portion, tumor located in upper portion. Bold values indicated p<0.05.

A logistic univariate analysis was performed for each variable in the derivation cohort (**Table 2A**). Male gender (OR, 1.855; 95% CI, 1.016–3.385; P = 0.044), maximum tumor diameter greater than 1.0 cm (OR, 4.378; 95% CI, 2.461–7.786; P <0.001), BRAFV600E mutation (OR, 2.976; 95% CI, 1.645–5.385; P <0.001), multifocality (OR, 3.270; 95% CI, 1.500–7.130; P = 0.003), the number of CLNM ≥3 (OR, 5.811; 95% CI, 3.265–10.343; P <0.001) and the ratio of CLNM ≥0.297 (OR, 6.403; 95% CI, 3.577–11.463; P <0.001) were statistically significantly. In addition, the incidence of LLNM among patients with prelaryngeal LNM (OR, 2.538; 95% CI, 1.138–5.662; P = 0.023) and pretracheal LNM (OR, 2.484; 95% CI, 1.447–4.264; P = 0.001) was significantly higher. Tumors located in the upper portion (OR, 2.112; 95% CI, 0.980–4.555; P = 0.056) were also recognized as independent risk factors for LLNM.

**Table 2A:** Univariate analysis of the derivation cohort.

Variables		N	LLNM (−), N = 132	LLNM (+), N = 96	OR (95% CI)	*P-*value
Gender	Female	170	105 (61.8)	65 (38.2)	1.855 (1.016–3.385)	**0.044**
	male	58	27 (46.6)	31 (53.4)		
Age	<55 years	200	116 (58.0)	84 (42.0)	1.036 (0.466–2.304)	0.931
	≥55 years	28	16 (57.1)	12 (42.9)		
HT	No	185	105 (56.8)	80 (43.2)	0.778 (0.393–1.540)	0.905
	Yes	43	27 (62.8)	16 (37.2)		
Calcification	No	206	119 (57.8)	87 (42.2)	0.947 (0.387–2.315)	0.905
	Yes	22	13 (59.0)	9 (41.0)		
Maximum tumor diameter	≤1 cm	146	103 (70.5)	43 (29.5)	4.378 (2.461–7.786)	**0.000**
	>1 cm	82	29 (35.5)	53 (64.5)		
Upper portion	No	197	119 (60.4)	78 (39.6)	2.112 (0.980–4.555)	0.056
	Yes	31	13 (41.9)	18 (58.1)		
BRAFV600E mutation	No	81	60 (74.1)	21 (25.9)	2.976 (1.645–5.385)	**0.000**
	Yes	147	72 (49.0)	75 (51.0)		
Nodular goiter	No	63	37 (58.7)	26 (41.3)	1.049 (0.582–1.890)	0.875
	Yes	165	95 (57.6)	70 (42.4)		
Multifocality	No	195	121 (62.1)	74 (37.9)	3.270 (1.500–7.130)	**0.003**
	Yes	33	11 (33.3)	22 (66.7)		
ETE	No	201	119 (59.2)	82 (40.8)	1.563 (0.698–3.498)	0.277
	Yes	27	13 (48.1)	14 (51.9)		
Prelaryngeal LNM	No	199	121 (60.8)	78 (39.2)	2.538 (1.138–5.662)	0.023
	Yes	29	11 (37.9)	18 (62.1)		
Pretracheal LNM	No	115	79 (68.7)	36 (31.3)	2.484 (1.447–4.264)	**0.001**
	Yes	113	53 (46.9)	60 (53.1)		
Paratracheal LNM	No	42	30 (71.4)	12 (28.6)	2.059 (0.993–4.268)	0.052
	Yes	186	102 (54.8)	84 (45.2)		
Number of CLNM ≥3	No	128	97 (75.8)	31 (24.2)	5.811 (3.265–10.343)	**0.000**
	Yes	100	35 (35.0)	65 (65.0)		
Ratio of CLNM ≥0.297	No	126	97 (77.0)	29 (23.0)	1.855 (1.016–3.385)	**0.000**
	Yes	102	35 (34.3)	67 (65.7)		

HT, Hashimoto thyroiditis; ETE, extrathyroidal extension; CLNM, central lymph node metastasis; LLNM, lateral lymph node metastasis; upper portion, tumor located in upper portion. Bold values indicated p<0.05. The symbol "(-)" indicated that no LLNM occurred. The symbol "(+)" indicated the occurrence of LLNM.

**Figure 3:**
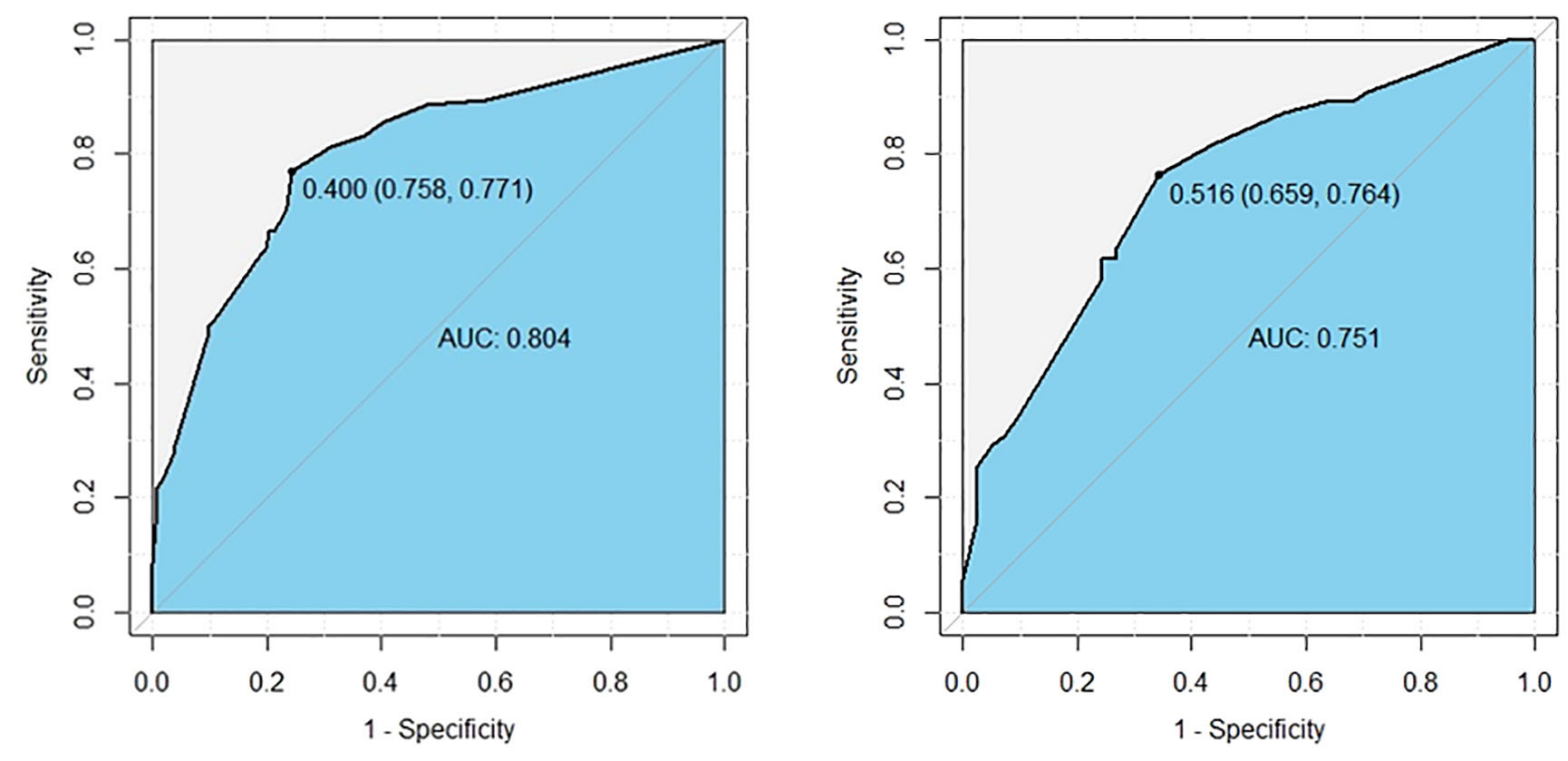
ROC curves show excellent discrimination ability for predicting LLNM in the derivation (left) and internal validation cohorts (right). The cutoff values were 0.400 (sensitivity: 0.758, specificity: 0.771), and 0.343 (sensitivity: 0.646, specificity: 0.744).

**Figure 4:**
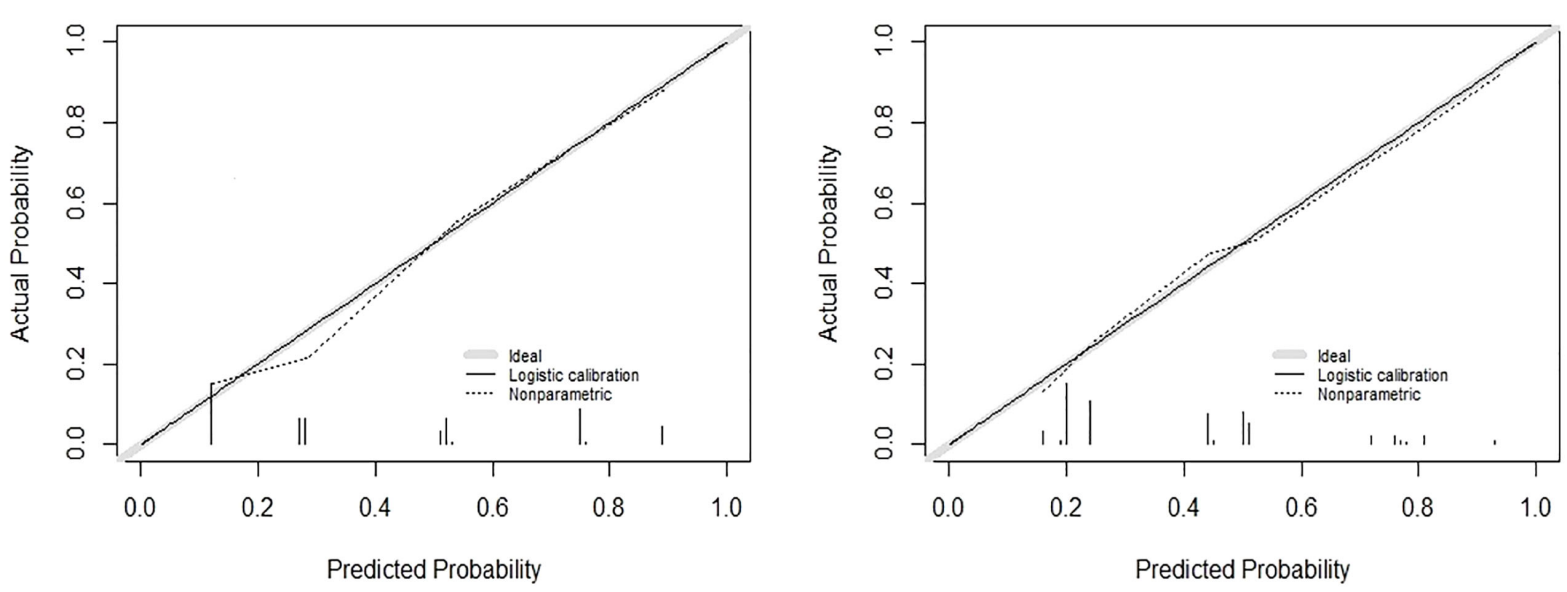
Calibration curves of the derivation cohort (left) and internal validation cohort (right).

The logistic multivariate analysis identified that maximum tumor diameter greater than 1.0 cm (OR, 2.712; 95% CI, 1.412–5.210; P = 0.003), multifocality (OR, 2.758; 95% CI, 1.120–6.789; P = 0.027), the number of CLNM ≥3 (OR, 2.579; 95% CI, 1.315–5.789; P = 0.007), the ratio of CLNM ≥0.297 (OR, 2.905; 95% CI, 1.396–6.043; P = 0.004) and tumor located in upper portion (OR 2.846, 95% CI 1.151–7.039, P = 0.024) were independent predictors of LLNM (**Table 2B**). A nomogram incorporating these five independent predictors was developed (**Figure 2**). Each predictor was assigned a point between 0 and 100 points. By summing the points and locating them on the total point scale, the corresponding probability of LLNM was determined. The ROC curve was generated, and the prediction model showed excellent discrimination ability with an AUC of 0.804 (95% CI, 0.745–0.863) (**Figure 3**). The calibration curve showed good agreement between the predicted and actual presence of LLNM, and the Hosmer–Lemeshow test indicated no departure from a good fit (P = 0.390) (**Figure 4**).

**Table 2B:** Multivariate analysis of the derivation cohort.

Independent predictors	β	Wald	OR (95% CI)	*P* value
Gender (male)	0.394	1.140	1.484 (0.719–3.061)	0.286
Maximum tumor diameter (>1 cm)	0.998	8.971	2.712 (1.412–5.210)	**0.003**
BRAF V600E (Yes)	0.249	0.433	1.283 (0.611–2.696)	0.511
Multifocality (Yes)	1.014	4.871	2.758 (1.120–6.789)	**0.027**
Prelaryngeal LNM (Yes)	0.150	0.090	1.162 (0.435–3.105)	0.764
Pretracheal LNM (Yes)	0.088	0.060	1.092 (0.539–2.213)	0.807
Number of CLNM ≥3 (Yes)	1.015	7.202	2.579 (1.315–5.789)	**0.007**
Ratio of CLNM ≥0.297 (Yes)	1.066	8.141	2.905 (1.396–6.043)	**0.004**
Upper portion (Yes)	1.046	5.127	2.846 (1.151–7.039)	**0.024**

Bold values indicated p<0.05.

**Figure 5:**
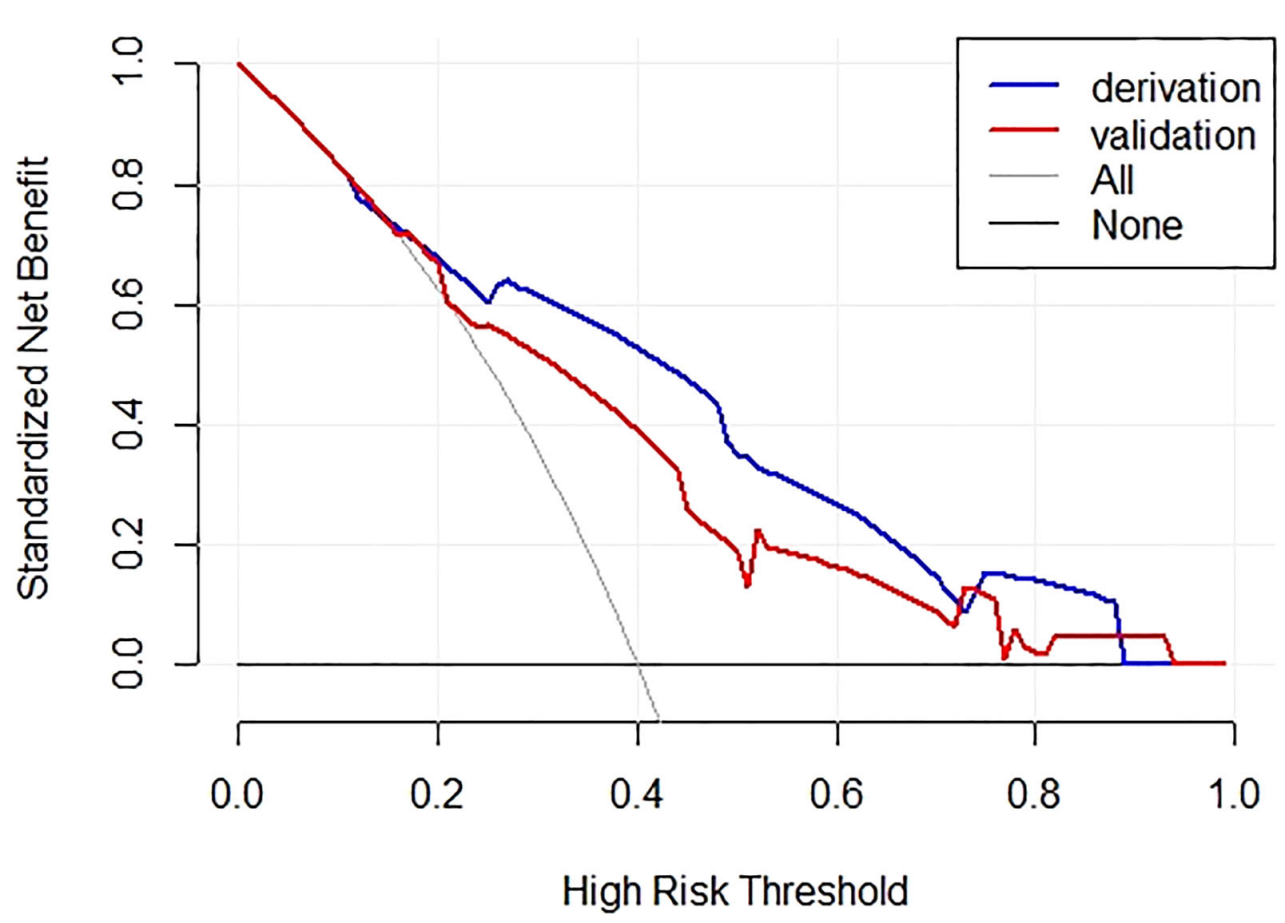
Decision curve of the derivation and validation cohorts.

An internal validation cohort was used to assess the predictive accuracy of the model. The ROC curve is shown in **Figure 3**, with an AUC of 0.731 (95% CI, 0.635–0.827). The excellent agreement between the actual and estimated probability of LLNM was displayed in the calibration curve (**Figure 4**), with a non-significant P = 0.339 in the Hosmer–Lemeshow test. The decision curve analysis revealed that if the threshold probability of LLNM was >10%, a greater net benefit would be presented by our nomogram (**Figure 5**). The cutoff value in the derivation cohort to distinguish the presence of LLNM was 0.400. The sensitivity, specificity, PPV, and NPV are listed in **Table 3**.

**Table 3:** The model performance in estimating the risk of LLNM.

Parameters	Derivation cohort	Internal validationcohort
Cutoff value	0.400	
AUC	0.804 (0.745–0.863)	0.731 (0.635–0.827)
Sensitivity	0.758 (0.672–0.848)	0.646 (0.510–0.781)
Specificity	0.771 (0.674–0.826)	0.744 (0.634–0.854)
PPV	0.698 (0.600–0.781)	0.604 (0.453–0.739)
NPV	0.820 (0.737–0.881)	0.767 (0.637–0.862)

PPV, Positive Predictive Value; NPV, Negative Predictive Value.

A novel risk stratification of LLNM was constructed based on this nomogram. The risk points of multifocality, maximum tumor diameter> 1 cm, number of CLNM ≥3, and tumors located in the upper portion were all 100 and the ratio of CLNM ≥0.297 was 110. Everyone could obtain total points by summing the risk points of each predictor. According to the distribution characteristics of LLNM prevalence, the cutoff value was chosen to stratify PTC patients into three subgroups: patients with total points = 0, 100, or 110 were assigned to the low-risk subgroup (probability of LLNM ≤20.4% (95% CI, 0.152–0.269)); patients with total points = 400, 410, and 510 were classified as the high-risk subgroup (probability of LLNM >60.2%); and the remaining patients were defined as the moderate-risk subgroup (20.4% <the probability of LLNM ≤60.2% (95% CI, 0.515–0.682)) (**Table 4A**). A significant difference was found among the three subgroups in terms of LLNM prevalence (**Table 4B**).

**Table 4A:** The incidence rate of LLNM in derivation and internal validation cohort patients with different total points.

Total points	LLNM		Total	The incidence rate of LLNM
	negative (−)	positive (+)		(LLNM+)/Total (%)
0, 100, 110	144	37	181	20.4
200, 210	29	37	66	56.1
300, 310	22	40	62	64.5
400, 410	2	24	26	92.3
510	0	1	1	100

According to the total points and corresponding incidence rate of LLNM, we stratified PTC patients into three subgroups: low-risk LLNM subgroup (total points = 0, 100, 110), moderate-risk subgroup (total points = 200, 210, 300, 310), and high-risk subgroup (total points = 400, 410, 510). We applied this risk stratification to the prospective validation cohort.

**Table 4B:** Risk stratification of patients.

Patients		LLNM		*P*-value
		negative (−)	positive (+)	
in retrospective cohort	low-risk	144	37	0.000
	moderate-risk	51	77	
	high-risk	2	25	
in prospective cohort	low-risk	25	12	0.000
	moderate-risk	15	31	
	high-risk	1	12	

### 3.2 The prospective validation

We used this risk stratification to explain the predictive LLNM risk stratification (**Table 4A**) to the patients before surgery. Combined with intraoperative frozen results, 37 (38.5%), 46 (47.9%), and 13 (13.5%) patients in the low-risk, moderate-risk, and high-risk subgroups, respectively underwent LLND (**Table 5**). **Figure 2** shows a sample case of the diagnostic use of this nomogram. Untimately, 96 newly diagnosed patients were included in the prospective validation cohort. The clinical characteristics of the cohort are summarized in **Table 1**. The incidence of prelaryngeal and pretracheal LNM in the prospective validation cohort was significantly higher than that in the retrospective cohort (P = 0.001 and 0.024, respectively). Tumors located in the upper portion and ETE were more commonly observed in the prospective validation cohort (P = 0.007 and 0.002, respectively). In addition, differences in the number and incidence of LLNM were obvious between the two cohorts (P = 0.008 and 0.026, respectively). Although no statistically significant difference in the distribution of risk subgroups was observed (P = 0.058), patients in the prospective validation cohort tended to have a higher LLNM risk stratification, which could be attributed to the application of this nomogram-based prediction model during surgery. In other words, this nomogram helped surgeons to efficiently screen high-risk patients with LLNM. In the prospective validation cohort, we found that the probability of LLNM was positively correlated with the risk points (linear fit: y = 540.2 × −65.19). The chi-square test revealed a significant difference among the three subgroups in terms of LLNM prevalence (**Table 4B**). These data confirmed that this prediction model is suitable for prospective clinical use.

**Table 5:** LLND preference of patients before surgery and actual intraoperative risk stratification.

LLND preference before surgery		No. of patients with CLNM	Intraoperative LLNM risk stratification		
LLNM risk stratification	No. of patients^1^		LLNM risk stratification	No. of patients^2^	Accepted LLND
≥low-risk	103	45	low-risk	37	√
			moderate-risk	7	√
			high-risk	1	√
≥moderate-risk	76	47	low-risk	5	
			moderate-risk	39	√
			high-risk	3	√
=high-risk	42	31	low-risk	8	
			moderate-risk	14	
			high-risk	9	√

^1^Number of patients who decided to undergo LLND according to the LLNM risk stratification before surgery.

^2^Number of patients who were stratified into three subgroups.

The symbol "√" indicated that LLND was conducted according to patient's preference before surgery.

## 4 Discussion

The development of LNM presents a stepwise pattern from the central to ipsilateral lateral compartments, except in some patients with PTC who have skip metastases (27). Although cN0 PTC patients still have a high incidence of occult CLNM (28), surgeons have disagreed on routine pCLND in view of related complications including parathyroid and laryngeal nerve injuries (29, 30). In fact, pCLND can provide information about tumor staging, guide postoperative radioiodine therapy (31) and improve the disease-free survival of patients with intermediate and high risks of structural recurrence (32). In our clinical practice, we performed routine pCLND for patients with PTC, and intraoperative frozen pathological results of CLNs were available. In our study, subjects in the derivation and internal validation cohorts were all cN0 PTC patients with CLNM, which indicated that pCLND could effectively screen occult CLNM. LLNM represents the involvement of level II–V cervical lymph nodes (33) and influences structural recurrence and prognostic outcome (34). Occult LLNM can occur in some patients with cN0 PTC and often remains undetected on preoperative imaging and palpation (35). However, pLLND was not recommended for these patients by most surgeons, considering postoperative complications such as bleeding, chyle leakage, and nerve injury (36). Some surgeons perform pLLND for high-risk PTC patients according to clinical features and experience, which is not sufficiently precise. Thus, it is necessary to confirm the risk factors for LLNM in cN0 PTC patients who might benefit from pLLND. According to the AJCC cancer staging manual, patients with PTC with CLNM (N1a) are more prone to LLNM (N1b) (37). In patients with cN0 PTC with CLNM, the incidence of occult LLNM was 41.4%. Patients with cN0 PTC have a low risk of skip metastasis (38). Thus, we selected cN0 PTC patients with CLNM for pLLND and analyzed the risk factors contributing to LLNM. Selective LLND (including levels II, III, and IV) was adopted as the extent of pLLND because it had similar recurrence rates to traditional LLND (levels II–V), and level V dissection might induce spinal accessory nerve injuries and shoulder dysfunction (35, 39).

Nomograms were used to visualize the prediction models and optimize the predictive accuracy (40, 41). Some studies had developed different nomograms based on clinical data to predict LLNM (42, 43). However, literature exploring prospective clinical use of a nomogram related to LLNM is lacking. We sought to explore the clinical application of this prediction model in eligible patients. We calculated the possibility of LLNM using this nomogram in the prospective validation group during surgery according to intraoperative data (tumor diameter, multifocality, tumor location, and central lymph node results) detected by frozen pathology. We succeeded in stratifying patients with PTC into three subgroups and identified high-risk patients with LLNM. Hence, our nomogram may be adequate for surgeons to evaluate the possibility of occult LLNM during surgery.

Several studies have elaborated that the number of CLNM was an essential risk factor for LLNM with cutoff values differed across these studies, ranging from 2 to 5 (44–47). CLNM ≥3 was identified as an independent risk factor for LLNM. Although recurrence was not observed in patients with cN0 PTC with 1–2 CLNM without LLND (13), patients with ≥3 CLNM should be screened for occult LLNM. The CLNM ratio is also considered a predictor of PTC prognosis (45). It was thought that the CLNM ratio was a risk factor for recurrence, and determining its cutoff value was helpful for identifying PTC patients with occult LLNM (48). We also identified the cutoff value of the CLNM ratio (≥0.297) for predicting LLNM in this study. In addition, larger tumor volume has been considered a significant risk factor for both CLNM and LLNM in patients with PTC (49–51). Our study found that PTC with a maximum tumor diameter greater than 1.0 cm tended to accompany LLNM. In addition, we found a relatively higher risk of LLNM involvement in multifocality, consistent with a previous study (10). Although it is still unclear whether multifocality is derived from multicentric lesions or intra-metastasis, the coexistence of multifocality suggests a more aggressive tumor. Moreover, we demonstrated that tumors located in the upper portion are prone to LLNM. It has been reported that PTC located in the upper portion has a distinctive lateral lymph node metastasis pattern (44, 52), and pLLND should be conducted more meticulously during surgery in cases of omission.

To explain the probability of occult LLNM more expediently, risk stratification was performed according to our nomogram. Intraoperative data were used to stratify patients and perform pLLND according to the patient’s preoperative wishes. The intraoperative frozen tissue data agreed well with the postoperative pathological results, indicating that our nomogram was accurate. For patients in the high-risk subgroup, the incidence of LLNM was 92.5%. Therefore, total thyroidectomy, pLLND, prophylactic adjuvant radioiodine (53) and TSH inhibition are recommended for high-risk patients with PTC. For patients in the moderate-risk subgroup, the incidence of LLNM was approximately 67.4% in the prospective cohort; pLLND was recommended, and an individualized treatment plan was made according to postoperative pathology. In the low-risk prospective subgroup, although the total incidence of LLNM was approximately 32.4%, the incidence was only 13.5% in patients with 0 points. Whether pLLND is performed in these patients depends on patient preference. There were no major postoperative complications in this prospective group, and only a few patients developed laboratory hypocalcemia, which was relieved with intravenous and oral calcium supplementation. Canu et al. (54) concluded that total thyroidectomy and hemithyroidectomy had no significant effect on the probability of postoperative complications. Moreover, a previous study showed that age is an important factor influencing the incidence of postoperative hypocalcemia, and timely calcium supplementation can reduce the probability of hypocalcemia (55).

Our study has several limitations. First, we constructed this prediction model retrospectively, which might have produced non-randomized features and a potential selection bias. Second, this study was conducted only at a single center, and postoperative follow-up is required. Third, this study only included cN0 unilateral PTC patients with CLNM to ensure the accuracy of model development. Therefore, patients with bilateral PTC should be included in future studies. A multicenter prospective study with a larger sample size is required to validate our nomogram.

## 5 Conclusion

In summary, we established a nomogram based on preoperative clinical and intraoperative pathological characteristics to predict the possibility of LLNM in patients with cN0 PTC with CLNM. This nomogram was applied in prospective clinical practice to distinguish PTC patients with a genuinely high risk of LLNM. It exhibits good discrimination and calibration abilities. Frozen pathology helped with intraoperative decisions regarding pLLND.

## Data availability statement

The raw data supporting the conclusions of this article will be made available by the authors, without undue reservation.

## Ethics statement

The studies involving humans were approved by the 960th Hospital of the PLA Joint Logistics Support Force Research Ethics Committee (No. 2022-57). The studies were conducted in accordance with local legislation and institutional requirements. All participants provided written informed consent to participate in the study.

## Author contributions

YHM: Conceptualization, Formal analysis, Funding acquisition, Investigation, Methodology, Project administration, Resources, Supervision, Writing – original draft, Writing – review & editing. YL: Conceptualization, Data curation, Formal analysis, Investigation, Methodology, Software, Validation, Visualization, Writing – original draft, Writing – review & editing. LMZ: Conceptualization, Formal analysis, Investigation, Supervision, Writing – review & editing. QQH: Conceptualization, Data curation, Funding acquisition, Investigation, Project administration, Resources, Writing – review & editing, Writing – original draft.

## References

[1] Siegel RLMK Fuchs HE Jemal A. Cancer statistics. CA Cancer J Clin (2022) 72(1):7–33. doi: 10.3322/caac.21708 35020204

[2] Xia CDX Li H Cao M Sun D He S Yang F. Cancer statistics in China and United States, 2022: profiles, trends, and determinants. Chin Med J (Engl) (2022) 135(5):584–90. doi: 10.1097/CM9.0000000000002108 PMC8920425 35143424

[3] Wang TS Sosa J. Thyroid surgery for differentiated thyroid cancer - recent advances and future directions. Nat Rev Endocrinol (2018) 14(11):670–83. doi: 10.1038/s41574-018-0080-7 30131586

[4] Chéreau NBC Trésallet C Tissier F Leenhardt L Menegaux F. Recurrence of papillary thyroid carcinoma with lateral cervical node metastases: Predictive factors and operative management. Surgery (2016) 159(3):755–62. doi: 10.1016/j.surg.2015.08.033 26435440

[5] Kim HI Kim T Choe JH Kim JH Kim JS Kim YN. Surgeon volume and prognosis of patients with advanced papillary thyroid cancer and lateral nodal metastasis. Br J Surg (2018) 105(3):270–8. doi: 10.1002/bjs.10655 29405275

[6] Hay ID Hutchinson M Gonzalez-Losada T Mciver B Reinalda ME Grant CS. Papillary thyroid microcarcinoma: a study of 900 cases observed in a 60-year period. Surgery (2008) 144(6):980–7. doi: 10.1016/j.surg.2008.08.035 19041007

[7] Lundgren CI Hall P Dickman PW Zedenius J. Clinically significant prognostic factors for differentiated thyroid carcinoma: a population-based, nested case-control study. Cancer Cytopathol (2006) 106(3):524–31. doi: 10.1002/cncr.21653 16369995

[8] Lee J Kim C Min IK Jeong S Kim H Choi MJ. Detailed characterization of metastatic lymph nodes improves the prediction accuracy of currently used risk stratification systems in N1 stage papillary thyroid cancer. Eur J Endocrinol (2020) 183(1):83–93. doi: 10.1530/EJE-20-0131 32487777

[9] Zhang F Russell Y Guber HA. Transverse and longitudinal ultrasound location of thyroid nodules and risk of thyroid cancer. Endocr Pract (2021) 27(7):682–90. doi: 10.1016/j.eprac.2021.01.009 33642256

[10] Haugen BR Alexander EK Bible KC Doherty GM Mandel SJ Nikiforov YE. 2015 American thyroid association management guidelines for adult patients with thyroid nodules and differentiated thyroid cancer: the american thyroid association guidelines task force on thyroid nodules and differentiated thyroid cancer. Thyroid (2016) 26(1):1–133. doi: 10.1089/thy.2015.0020 26462967 PMC4739132

[11] Ito Y Onoda N Okamoto T. The revised clinical practice guidelines on the management of thyroid tumors by the Japan Associations of Endocrine Surgeons: Core questions and recommendations for treatments of thyroid cancer. Endocr J (2020) 67(7):669–717. doi: 10.1507/endocrj.EJ20-0025 32269182

[12] AR S. Prophylactic central compartment dissection in thyroid cancer: a new avenue of debate. Surgery (2009) 146(6):1224–7. doi: 10.1016/j.surg.2009.10.020 19958952

[13] Wang Y Deng C Shu X Yu P Wang H Su X. Risk factors and a prediction model of lateral lymph node metastasis in CN0 papillary thyroid carcinoma patients with 1-2 central lymph node metastases. Front Endocrinol (Lausanne) (2021), 12:716728. doi: 10.3389/fendo.2021.716728 34721289 PMC8555630

[14] Nixon IJ Wang LY Ganly I Patel SG Morrise LG Migliacci JC. Outcomes for patients with papillary thyroid cancer who do not undergo prophylactic central neck dissection. Br J Surg (2016) 103(3):218–25. doi: 10.1002/bjs.10036 PMC4976488 26511531

[15] de Meer SG Dauwan M De Keizer B Valk GD Borel Rinkes IH Vriens MR. Not the number but the location of lymph nodes matters for recurrence rate and disease-free survival in patients with differentiated thyroid cancer. World J Surg (2012) 36(6):1262–7. doi: 10.1007/s00268-012-1427-1 PMC3348473 22270993

[16] Mulla MG Knoefel W Gilbert J Mcgregor A Schulte KM. Lateral cervical lymph node metastases in papillary thyroid cancer: a systematic review of imaging-guided and prophylactic removal of the lateral compartment. Clin Endocrinol (Oxf) (2012) 77(1):126–31. doi: 10.1111/j.1365-2265.2012.04336.x 22233478

[17] Patron V Bedfert C Le Clech G Aubry K Jegoux F. Pattern of lateral neck metastases in N0 papillary thyroid carcinoma. BMC Cancer (2011) 11:8. doi: 10.1186/1471-2407-11-8 21223538 PMC3023783

[18] Zhan S Luo D Ge W Zhang B Wang T. Clinicopathological predictors of occult lateral neck lymph node metastasis in papillary thyroid cancer: A meta-analysis. Head Neck (2019) 41(7):2441–9. doi: 10.1186/1471-2407-11-8 30938923

[19] Collins GS Reitsma J Altman DG Moons KG. Transparent reporting of a multivariable prediction model for individual prognosis or diagnosis (TRIPOD): the TRIPOD statement. BMJ (2015) 7:350. doi: 10.1136/bmj.g7594 25569120

[20] Zhang Z Zhang X Yin Y Zhao S Wang K Shang M. Integrating BRAFV600E mutation, ultrasonic and clinicopathologic characteristics for predicting the risk of cervical central lymph node metastasis in papillary thyroid carcinoma. BMC Cancer (2022) 22(1):461. doi: 10.1186/s12885-022-09550-z 35473554 PMC9044661

[21] Zhu S Wang Q Zheng D Zhu L Zhou Z Xu S. A novel and effective model to predict skip metastasis in papillary thyroid carcinoma based on a support vector machine. Front Endocrinol (Lausanne) (2022) 13:916121. doi: 10.3389/fendo.2022.916121 35865315 PMC9295388

[22] Kobaly K Kim CS Mandel SJ. Contemporary management of thyroid nodules. Annu Rev Med (2022) 73(1):517–28. doi: 10.1146/annurev-med-042220-015032 34416120

[23] Park JY Lee H Jang HW Kim HK Yi JH Lee W. A proposal for a thyroid imaging reporting and data system for ultrasound features of thyroid carcinoma. Thyroid (2009) 19(11):1257–64. doi: 10.1089/thy.2008.0021 19754280

[24] Chen M-X Meng X-Q Zhong Z-H Tang XJ Li T Feng Q. An individualized recommendation for controlled ovary stimulation protocol in women who received the gnRH agonist long-acting protocol or the gnRH antagonist protocol: A retrospective cohort study. Front Endocrinol (2022) 13:899000. doi: 10.3389/fendo.2022.899000 PMC9355571 35937797

[25] Kim K Kim J Park IS Rho YS Kwon GH Lee DJ. The updated AJCC/TNM staging system for papillary thyroid cancer (8th edition): from the perspective of genomic analysis. World J Surg (2018) 42(11):3624–31. doi: 10.1007/s00268-018-4662-2 29750323

[26] Mathew G Agha R Albrecht J Goel P Mukherjee I Pai P. STROCSS 2021: Strengthening the reporting of cohort, cross-sectional and case-control studies in surgery. Int J Surg (2021) 96:106165. doi: 10.1016/j.ijsu.2021.106165 34774726

[27] Xue S Wang P Liu J Li R Zhang L Chen G. Prophylactic central lymph node dissection in cN0 patients with papillary thyroid carcinoma: A retrospective study in China. Asian J Surg (2016) 39(3):131–6. doi: 10.1016/j.asjsur.2015.03.015 26117203

[28] Mazzaferri EL Doherty G Steward DL. The pros and cons of prophylactic central compartment lymph node dissection for papillary thyroid carcinoma. Thyroid (2009) 19(7):683–9. doi: 10.1089/thy.2009.1578 19583485

[29] S. AV. Optimization of staging of the neck with prophylactic central and lateral neck dissection for papillary thyroid carcinoma. Ann Surg (2015) 261(1):e30. doi: 10.1097/SLA.0000000000000510 24441800

[30] De Napoli L Matrone A Favilla K Piaggi P Galleri D Ambrosini CE. Role of prophylactic central compartment lymph node dissection on the outcome of patients with papillary thyroid carcinoma and synchronous ipsilateral cervical lymph node metastases. Endocr Pract (2020) 26(8):807–17. doi: 10.4158/EP-2019-0532 33471672

[31] Medas F Canu G Cappellacci F Anedda G Conzo G Erdas E. Prophylactic central lymph node dissection improves disease-free survival in patients with intermediate and high risk differentiated thyroid carcinoma: A retrospective analysis on 399 patients. Cancers (Basel) (2020) 12(6):1658. doi: 10.3390/cancers12061658 32585797 PMC7353019

[32] Wada N Duh Q Sugino K Iwasaki H Kameyama K Mimura T. Lymph node metastasis from 259 papillary thyroid microcarcinomas: frequency, pattern of occurrence and recurrence, and optimal strategy for neck dissection. Ann Surg (2003) 237(3):399–407. doi: 10.1097/01.SLA.0000055273.58908.19 12616125 PMC1514312

[33] Nixon IJ Wang L Palmer FL Tuttle RM Shaha AR Shah JP. The impact of nodal status on outcome in older patients with papillary thyroid cancer. Surgery (2014) 156(1):137–46. doi: 10.1016/j.surg.2014.03.027 24878458

[34] Lim YS Lee J Lee YS Lee BJ Wang SG Son SM. Lateral cervical lymph node metastases from papillary thyroid carcinoma: predictive factors of nodal metastasis. Surgery (2011) 150(1):116–21. doi: 10.1016/j.surg.2011.02.003 21507446

[35] Roh JL Kim JM P. CI. Lateral cervical lymph node metastases from papillary thyroid carcinoma: pattern of nodal metastases and optimal strategy for neck dissection. Ann Surg Oncol (2008) 15(4):1177–82. doi: 10.1245/s10434-008-9813-5 18253801

[36] Edge SB Compton C. The American Joint Committee on Cancer: the 7th edition of the AJCC cancer staging manual and the future of TNM. Ann Surg Oncol (2010) 17(6):1471–4. doi: 10.1245/s10434-010-0985-4 20180029

[37] Hu D Lin H Zeng X Wang T Deng J Su X. Risk factors for and prediction model of skip metastasis to lateral lymph nodes in papillary thyroid carcinoma. World J Surg (2020) 44(5):1498–505. doi: 10.1007/s00268-019-05332-0 31863139

[38] Won HR Chang JW Kang YE Kang JY Koo BS. Optimal Extent of Lateral dissection for well-differentiated thyroid carcinoma with metastatic lateral neck lymph nodes A systematic review and meta-analysis. Oral Oncol (2018) 87:117–25. doi: 10.1016/j.oraloncology.2018.10.035 30527226

[39] Balachandran VP Gonen M Smith JJ DeMatteo RP. Nomograms in oncology: more than meets the eye. Lancet Oncol (2015) 16:e173–80. doi: 10.1016/S1470-2045(14)71116-7 PMC4465353 25846097

[40] Chang Q Zhang J Wang Y Li H Du X Zuo D. Nomogram model based on preoperative serum thyroglobulin and clinical characteristics of papillary thyroid carcinoma to predict cervical lymph node metastasis. Front Endocrinol (Lausanne) (2022) 13:937049. doi: 10.3389/fendo.2022.937049 35909521 PMC9337858

[41] Thompson AM Turner R Hayen A Aniss A Jalaty S Learoyd DL. A preoperative nomogram for the prediction of ipsilateral central compartment lymph node metastases in papillary thyroid cancer. Thyroid (2014) 24(4):675–82. doi: 10.1089/thy.2013.0224 PMC3993080 24083952

[42] Dai Q Liu D Tao Y Ding C Li S Zhao C. Nomograms based on preoperative multimodal ultrasound of papillary thyroid carcinoma for predicting central lymph node metastasis. Eur Radiol (2022) 32(7):4596–608. doi: 10.1007/s00330-022-08565-1 35226156

[43] Heng Y Yang Z Zhou L Lin J Cai W Tao L. Risk stratification for lateral involvement in papillary thyroid carcinoma patients with central lymph node metastasis. Endocrine (2020) 68(2):320–8. doi: 10.1007/s12020-020-02194-8 31983029

[44] Lee YS Lim Y Lee JC Wang SG Kim IJ Lee BJ. Clinical implication of the number of central lymph node metastasis in papillary thyroid carcinoma: preliminary report. World J Surg (2010) 34(11):2558–63. doi: 10.1007/s00268-010-0749-0 20703463

[45] Liu S Liu C Zhao L Wang K Li S Tian Y. A prediction model incorporating the BRAF(V600E) protein status for determining the risk of cervical lateral lymph node metastasis in papillary thyroid cancer patients with central lymph node metastasis. Eur J Surg Oncol (2021) 47(11):2774–80. doi: 10.1016/j.ejso.2021.08.033 34483032

[46] Rajeev P Ahmed S Ezzat TM Sadler GP Mihai R. The number of positive lymph nodes in the central compartment has prognostic impact in papillary thyroid cancer. Langenbecks Arch Surg (2013) 398(3):377–82. doi: 10.1007/s00423-012-1041-6 23274361

[47] Vas Nunes JH Clark J Gao K Chua E Campbell P Niles N. Prognostic implications of lymph node yield and lymph node ratio in papillary thyroid carcinoma. Thyroid (2013) 23(7):811–6. doi: 10.1089/thy.2012.0460 23373961

[48] Wu X Li B Zheng C He X. Predicting factors of lateral neck lymph node metastases in patients with papillary thyroid microcarcinoma. Med (Baltimore) (2019) 98(27):e16386. doi: 10.1097/MD.0000000000016386 PMC6635253 31277195

[49] Feng JW Qin AC Ye J Pan H Jiang Y Qu Z. Predictive factors for lateral lymph node metastasis and skip metastasis in papillary thyroid carcinoma. Endocr Pathol (2020) 31(1):67–76. doi: 10.1007/s12022-019-09599-w 31828583

[50] KIM J Gosnell J ROMAN SA. Geographic influences in the global rise of thyroid cancer. Nat Rev Endocrinol (2020) 16(1):17–29. doi: 10.1038/s41574-019-0263-x 31616074

[51] Qu HJ Qu X Hu Z Lin Y Wang JR Zheng CF. The synergic effect of BRAFV600E mutation and multifocality on central lymph node metastasis in unilateral papillary thyroid carcinoma. Endocr J (2018) 65(1):113–20. doi: 10.1507/endocrj.EJ17-0110 29070763

[52] Likhterov I Reis L Urken ML. Central compartment management in patients with papillary thyroid cancer presenting with metastatic disease to the lateral neck: Anatomic pathways of lymphatic spread. Head Neck (2017) 39(5):853–9. doi: 10.1002/hed.24568 28252836

[53] H BR. 2015 American Thyroid Association Management Guidelines for Adult Patients with Thyroid Nodules and Differentiated Thyroid Cancer: What is new and what has changed? Cance (2017) 123(3):372–81. doi: 10.1002/cncr.30360 27741354

[54] Canu GL Medas F Cappellacci F Giordano ABF Gurrado A Gambardella C. Risk of complications in patients undergoing completion thyroidectomy after hemithyroidectomy for thyroid nodule with indeterminate cytology: an italian multicentre retrospective study. Cancers (Basel). (2022) 14(10):2472. doi: 10.3390/cancers14102472 35626075 PMC9139447

[55] Tolone S Roberto R del Genio G Brusciano L Parmeggiani D Amoroso V. The impact of age and oral calcium and vitamin D supplements on postoperative hypocalcemia after total thyroidectomy. A prospective study. BMC Surg (2013) 13 Suppl2(Suppl 2):S11. doi: 10.1186/1471-2482-13-S2-S11 24267491 PMC3851172

